# Clinical Potentials of Cardiomyocytes Derived from Patient-Specific Induced Pluripotent Stem Cells

**DOI:** 10.3390/jcm3041105

**Published:** 2014-10-15

**Authors:** Kwong-Man Ng, Cheuk-Yiu Law, Hung-Fat Tse

**Affiliations:** 1 Cardiology Division, Department of Medicine, Rm. 1928, Block K, Queen Mary Hospital, the University of Hong Kong, Hong Kong SAR, China; E-Mails: skykmng@hkucc.hku.hk (K.-M.N.); cylaw1@hotmail.com (C.-Y.L.); 2 Research Center of Heart, Brain, Hormone and Healthy Aging, Li Ka Shing Faculty of Medicine, the University of Hong Kong, Hong Kong SAR, China; 3 Hong Kong-Guangdong Joint Laboratory on Stem Cell and Regenerative Medicine, the University of Hong Kong and Guangzhou Institutes of Biomedicine and Health, Hong Kong SAR, China; 4 Shenzhen Institutes of Research and Innovation, the University of Hong Kong, Hong Kong SAR, China

**Keywords:** patient-specific-iPSCs-derived cardiomyocytes, regenerative medicine, modeling of inherited cardiac disorder, drug testing, toxicology studies

## Abstract

The lack of appropriate human cardiomyocyte-based experimental platform has largely hindered the study of cardiac diseases and the development of therapeutic strategies. To date, somatic cells isolated from human subjects can be reprogramed into induced pluripotent stem cells (iPSCs) and subsequently differentiated into functional cardiomyocytes. This powerful reprogramming technology provides a novel *in vitro* human cell-based platform for the study of human hereditary cardiac disorders. The clinical potential of using iPSCs derived from patients with inherited cardiac disorders for therapeutic studies have been increasingly highlighted. In this review, the standard procedures for generating patient-specific iPSCs and the latest commonly used cardiac differentiation protocols will be outlined. Furthermore, the progress and limitations of current applications of iPSCs and iPSCs-derived cardiomyocytes in cell replacement therapy, disease modeling, drug-testing and toxicology studies will be discussed in detail.

## 1. Introduction

Cardiomyocytes, or heart muscle cells, are fragile but important constituents of the myocardium. It is generally believed that humans are born with a fixed amount of cardiomyocytes; therefore, the death of these muscle cells may cause permanent damage to the heart. Recently, Bergmann and colleagues have evidenced a revolutionary notion of the *in vivo* regeneration and renewal of cardiomyocytes in humans [1]; nevertheless, the rate of cardiomyocyte turnover in their experiment appeared to be extremely slow. In fact, following myocardial injury, the heart usually repairs itself by cellular hypertrophy [2]. In case of a substantial loss of cardiomyocytes such as severe myocardial infarction, the damaged tissue is replaced with fibroblasts, rather than functional cardiomyocytes. To this end, the heart function is permanently impaired. Attempts of using adult stem cells or embryonic stem cells in replacing the damaged myocardium have been made, and several successful cases have been reported. Yet, such a replacement approach is impeded by various factors, for instance, the limiting sources of stem cells as well as the non-self rejection issues. In 2007, Yamanaka and colleagues demonstrated the first time that adult human fibroblasts could be reprogrammed into the pluripotent stem cells when supplemented with well-defined culturing factors [3]. Based on this revolutionary reprogramming approach, any fully differentiated cells obtained from patients should be theoretically able to be reprogrammed into induced pluripotent stem cells (iPSCs), and further differentiated into specialized cells of desired interest such as cardiac derivatives. The iPSCs obtained would be patient-specific; they not only provide a new source for regenerative medicine, but also offer a human cell based platform for the studies of modeling of inherited cardiac diseases and screening of potential cardiovascular drugs. In this review, the clinical potentials of patient-specific iPSCs in therapeutic treatments of cardiac disorders will be addressed in detail.

## 2. Patient-Specific iPSCs and Their Cardiac Derivatives

**Figure 1:**
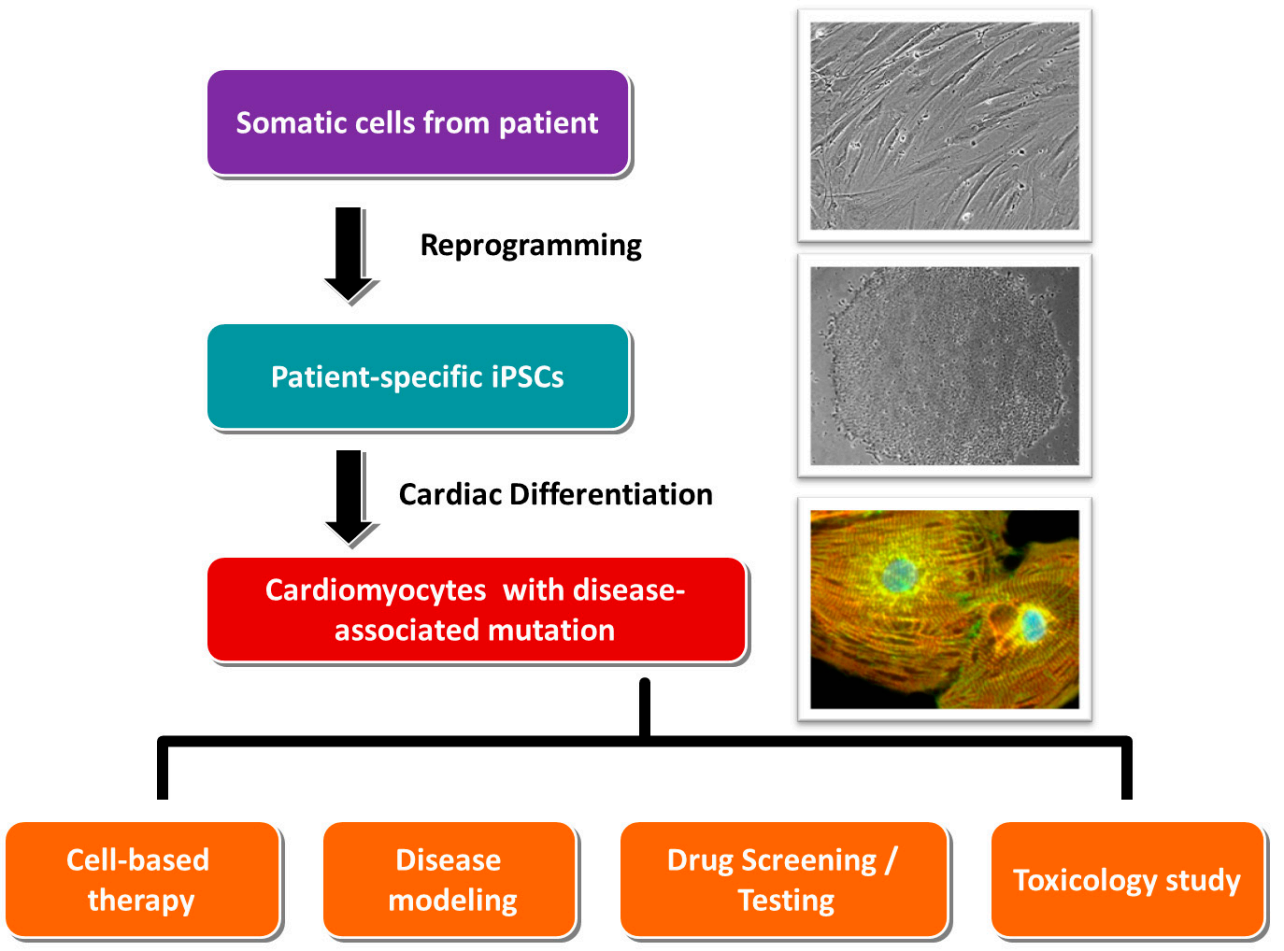
The clinical applications of the cardiomyocytes derived from patient-specific iPSCs.

In 2006, Yamanaka and colleagues demonstrated for the first time that the exogenous expression of four transcription factors—Oct4, Klf-4, Sox-2 and c-Myc [4]—Initiated the reprogramming of terminally differentiated murine somatic cells (skin fibroblasts) into iPSCs, which were characterized with adequate pluripotency. Similar to embryonic stem cells, these iPSCs were able to self-renew, proliferate and differentiate into various cell types including neurons and cardiomyocytes [5,6]. The same research group at a later time showed that human somatic cells could also be reprogrammed into iPSCs [3,7]. These technological breakthroughs have made substantial impacts in cell replacement therapy, disease modeling and therapeutic discovery sectors. Although the cells from a patient with myocardial infarction can be reprogrammed and differentiated into functional cardiomyocytes, the replacement of the defective cells of a particular patient is still theoretical. Nevertheless, iPSCs generated from patients with inherited cardiac diseases, following *in vitro* cardiac differentiation, are still valuable tools for disease modeling and development of personalized medicine (Figure 1), as the iPSCs-derived cardiomyocytes possess the defective genes of the patients.

## 3. Standard Procedures in Generating Patient-Specific iPSCs and Their Cardiac Derivatives

In general, the generation of human iPSCs-derived cardiomyocytes involves three major steps: (i) collection of somatic tissues/cells; (ii) reprogramming; and (iii) cardiac differentiation.

### 3.1. Collection of Somatic Tissues/Cells

The protocol of Yamanaka and colleagues suggested the use of skin fibroblasts as the starting material of iPSCs generation. However, the invasive procedures of collecting skin biopsy actually caused many patients, especially pediatric subjects, to refuse donating tissue samples for iPSCs generation. In this regard, less invasive alternatives are obviously more preferable in clinical practices. It is now evidenced that apart from skin fibroblasts, many other cell types, such as hair follicle cells, peripheral blood cells as well as uro-epithelial cells, could also be reprogrammed into iPSCs [8,9,10,11,12,13]. Among these cells, the collection of uro-epithelial cells from urine accounts for the simplest and most convenient way. This non-invasive method eliminates pain or wound caused by skin biopsy collection; thus, is more likely to be accepted by patients. In fact, our laboratory is now routinely collecting urine samples from patients for iPSCs generation [14,15].

### 3.2. Reprogramming

The first generation of reprogramming method involved the use of retrovirus vectors in infecting four transcription factors (Oct4, Klf4, Sox2 and c-Myc) into cultured fibroblasts. This method is quite robust; for this reason, many laboratories, including ours, are routinely using this method for iPSCs generation. However, the reprogramming efficiency of this method is not high (about 0.0002%). Moreover, the use of retrovirus vectors is a big concern in clinical applications. Therefore, several alternative methods have been proposed. For example, Nanog and Lin28 are suggested as additional reprogramming factors in some protocols since the addition of these two factors increased the efficiency to about 0.05% for fetal fibroblasts [16]. As retrovirus only infects actively dividing cells, the use of lentivirus-based vectors may be a better option for the cell types that are non-actively dividing or less proliferative (e.g., cardiac fibroblasts). It is generally accepted that lentivirus-based vectors can transduce both dividing and non-dividing cells. On top of that, lentivirus-based vectors can accommodate much larger inserts. The four essential reprogramming factors Oct4, Klf4, Sox2 and c-Myc could be linked up within one single expression cassette [17] and simultaneously inserted into a single vector. This strategy eliminates the need for producing multiple transducing vectors; thus it avoids the possible stoichiometric and temporal interference among individual viruses [18].

Despite that retrovirus- and lentivirus-based vectors are widely used in iPSCs generation, the incorporation of viral sequences into the host genome is still an important concern in clinical applications, especially in the cell replacement therapy utilizing patient-specific iPSCs. For addressing this issue, the application of a Cre-loxP system in the lentivirus backbone has been suggested, so that the viral sequences could be eventually cleaved from the host genome upon the execution of Cre-recombinase [19,20]. Nevertheless, the use of non-integrative viruses appears to be a more acceptable method. For example, non-integrative viruses, such as adenovirus and Sendai virus, have been successfully used in some reprogramming protocols of human fibroblasts [21,22]. In addition, epigenetic reprogramming methods, such as transfection of mRNA, miRNA, minicircle vectors and episomal plasmids are regarded as the possible alternatives for footprint-free iPSCs reprogramming.

### 3.3. Cardiac Differentiation

In spontaneous differentiation, cardiomyocyte is one of the most easily identifiable cell types. Even in the absence of specific growth factors, spontaneous beating clusters could be observed when iPSCs are allowed to form aggregates (embryoid bodies) in a culturing suspension. However, the actual number of cardiomyocytes within a spontaneous beating embryoid body may comprise as low as merely 1% of the total cell population. It is obvious that spontaneous differentiation is not sufficient for the generation of iPSCs-derived cardiomyocytes in an adequate quantity for most experimental assays or applications. Early methods of directing cardiac differentiation involved the co-culture of iPSCs with END-2 endodermal cells. This END-2 co-culturing method has been used widely for cardiac differentiation of human embryonic stem cells and is relatively robust [23,24]. However, the difficulty in separating the cardiomyocytes from the feeder layers denotes a main drawback of this method. To date, many feeder-free cardiac differentiation protocols have been developed, so that problems associated with feeder layers can be eliminated. Most of these feeder-free methods involve the incubation with growth factors, such as the BMP4, Activin A, VEGF and DKK that regulate the pathways directing heart formation during fetal developments [6,25]. Recently, Palecek and colleagues reported a successful application of Wnt pathway inhibitors in directing cardiac differentiation of human iPSCs [26,27]. By modulating the Wnt/β-catenin signaling under fully defined conditions, monolayers of virtually pure cardiomyocytes (up to 98%) were obtained in merely 14 days.

## 4. Application of Patient-Specific iPSCs in Cell Replacement Therapy/Regenerative Medicine

Increasing evidences showed that the adult human heart possesses a certain degree of regenerating power. Following severe cardiac injury, cardiac hypertrophy and scarring are indeed the major repairing mechanisms to maintain minimum cardiac functions and prevent further damages. However, without the replacement of new cardiomyocytes, the ordinary repairing mechanisms usually result in the continual increase of cardiac workload that further worsens the injured condition and even leads to chronic heart failure of the patient. To this end, various studies have been converged on the use of pluripotent stem cells in cardiac recovery.

A previous study reported that the transplantation of human embryonic stem cells-derived cardiomyocytes into the infarcted myocardium of an immunodeficient rodent partially remuscularized myocardial infarcts and improved cardiac function [28,29]. At a later stage, Gaballa and colleagues demonstrated that cell sheets composed of rat or human cardiac progenitor cells, when transplanted into the infarcted heart, could proliferate and differentiate into functional cardiomyocytes, and rescue myocardial function [30].

Besides human embryonic stem cells, human iPSCs also possess the ability to differentiate into cardiac lineages. Since iPSCs can be derived from any individual, the use of human iPSCs in regenerative medicine can avoid the ethical issues arising from the use of embryonic materials. Furthermore, iPSCs can be produced from the same individual who is receiving the cell replacement therapy, so that immunological incompatibility should become less significant.

Recently, Watt and colleagues attempted to investigate the potential benefits of human iPSCs-derived progenitors. In their study, human iPSCs-derived cardiac progenitor cells were injected into the pre-infarct hearts of rats. The injected cells were able to differentiate into cardiomyocytes and smooth muscle fibers, and were retained in the rat hearts for at least 10 weeks after myocardial infarction. When comparing to the control group, the animals that received human-iPSCs-derived cardiac progenitor cells showed some improvement in the left ventricular ejection fraction [31].

It should be noted that although many studies have pointed out the beneficial effects of human embryonic stem cells and iPSCs against ischemic cardiac injuries, most of the studies only involved a relatively short follow-up period (see Table 1); thus, the long term efficacy of iPSCs-derived cardiac progenitors remains questionable.

**Table 1:** Examples of using iPSCs in cell replacement therapies.

Cell Type	Animal Model	Number of Cell	Delivery Method	Timing of the Delivery	Follow up Duration	Reference
iPSC	Mouse	50,000	IM	Immediately after MI induction	2 weeks	[30]
iPSC-derived cardiac progenitors	Rat	2 × 10^6^	IM	10 min after MI induction	10 weeks	[31]
Cardiosphere	Rat	-	Cell sheet	Immediately after MI induction	3 weeks	[32]

## 5. Applications of iPSCs-Derived Cardiomyocytes in Modeling Genetic Cardiomyopathies

Cardiomyopathies are heterogeneous groups of diseases of cardiomyocytes. Pathologically, the diseases could be caused by non-genetic factors such as viral infection though genetic contributions are frequently observed [33]. To date, over 50 genes have been reported to be associated with various forms of cardiomyopathies; yet, the studies of the pathogenic mechanisms underlying specific genetic defects remain elusive due to the lack of appropriate experimental models.

Theoretically, the affected tissues obtained from patients with cardiomyopathies are the best options for pathophysiological studies; however, for cardiac diseases, the limitation in obtaining and maintaining cardiac biopsy samples highly hindered this strategy.

To compensate this limitation, transgenic animals are commonly used for modeling human genetic defects. Nonetheless, due to the substantial physiological differences between the hearts of human and those of mice [34,35,36], the use of transgenic mouse lines in modeling human genetic cardiomyopathies is of little practical value. For example, in terms of ion-channel physiology, transgenic mouse models, in most cases, only partly recapitulate the disease phenotypes [36,37]. The phenotypic differences between species accentuate the importance of a novel human cardiomyocyte-based model in the studies of heritable cardiac defects, and the cardiomyocytes derived from patient-specific iPSCs should be one of the most desirable options.

In general, if the mutation of the gene of interest does not interfere in cardiac differentiation, cardiomyocytes can be continually generated from patient-specific iPSCs. This continuous supply of cardiomyocytes indeed resembles the cardiac biopsy samples that could hardly be obtained from patients with specific inherited cardiac defects; thus these patient-specific iPSCs-derived cardiomyocytes provide a convenient and valuable platform for research purposes. In fact, various recent reports have demonstrated that the cardiomyocytes derived from patient-specific iPSCs were able to recapitulate disease phenotypes of various types of Long QT syndromes [38,39,40]. These data clearly evidenced the feasibility of utilizing the patient-specific iPSCs-derived cardiomyocytes in modeling heritable cardiomyopathies.

In 2010, Laugwitz and colleagues established the first patient-specific iPSCs based model for type 1 Long QT syndrome [41]. In their study, the skin fibroblasts from patients carrying an autosomal dominant missense mutation (R190Q) in the *KCNQ1* gene were effectively reprogrammed into iPSCs. The resultant iPSCs were further differentiated into atrial- and ventricular-like cardiomyocytes and subjected to patch-clamp analysis. When comparing to the control, the iPSCs-derived cardiomyocytes with the *KCNQ1* mutation showed a markedly prolonged duration of action potential, altered activation and deactivation properties of IKs, and an abnormal response to catecholamine stimulation. Immunostaining analysis demonstrated the failure of mutated K_v_7.1 potassium channel protein in its trafficking to the plasma membrane; this finding may provide an explanation to the cellular pathogenic mechanism of the *KCNQ1*_R190Q_ mutation. Undoubtedly, the use of iPSCs-derived cardiomyocytes in modeling inherited cardiac disorders is feasible.

In addition to Long QT syndromes, cardiomyocytes derived from patient-specific iPSCs have also been used in the modeling of some other genetic cardiac disorders (Table 2); examples are outlined as follows:

**Table 2:** Examples of using iPSCs-derived cardiomyocytes for modeling genetic cardiomyopathies.

Disorder	Gene Involved	Details of the Mutation	References
Long QT syndrome, Type 1	*KCNQ1*	missense mutation (R190Q) leads to the production of a mutant protein	[41]
Long QT syndrome, Type 2	*KCNH2*	missense mutation (A614V) leads to the production of a mutant protein	[38]
Long QT syndrome, Type 2	*KCNH2*	missense mutation (G1618A) leads to the production of a mutant protein	[40]
Long QT syndrome, Type 2	*KCNH2*	missense mutation (R176W) leads to the production of mutant protein	[42]
Long QT syndrome, Type 3	*SCN5A*	Multiple mutations (G5287A; V1763M) leads the production of a mutant protein	[43]
Long QT syndrome, Type 8	*CACNA1C*	Missense mutation (G406R) leads to the production of a mutant protein	[44]
Catecholaminergic polymorphic ventricular tachycardia, Type 1	*RYR2*	Missense mutation (F2483I) leads to the production of a mutant protein with an altered FKBP12.6 binding domain	[45]
Catecholaminergic polymorphic ventricular tachycardia, Type 1	*RYR2*	Missense mutation (S406L) leads to the production of a mutant protein	[46]
Catecholaminergic polymorphic ventricular tachycardia, Type 2	*CASQ2*	Missense mutation (D307H) leads to the production of a mutant protein	[47]
Catecholaminergic polymorphic ventricular tachycardia, Type 2	*CASQ2*	Missense mutation (D307H) leads to the production of a mutant protein	[47]
Dilated cardiomyopathy	*TNNT2*	missense mutation (R173W) leads to the production of a mutant protein	[48]
Dilated cardiomyopathy	*DES*	missense mutation (A285V) leads to the production of a mutant protein	[49]
Hypertrophic cardiomyopathy	*MYH7*	Missense mutation (R663H) leads to the production of a mutant protein	[50]
Friedreich ataxia-associated hypertrophic cardiomyopathy	*FXN*	GAA repeat expansion in the first intron leads to the partial silencing of gene expression	[51]

### 5.1. Modeling Catecholaminergic Polymorphic Ventricular Tachycardia (CPVT)

Although inherited arrhythmogenic disorders are frequently associated with the mutations in the genes encoding the ion channel components, a special kind of inherited ventricular arrhythmia called catecholaminergic polymorphic ventricular tachycardia (CPVT) is caused by the mutations in genes encoding the proteins mediating intracellular calcium transient. In response to emotional or physical stress, CPVT patients may manifest ventricular premature beats and bidirectional or polymorphic ventricular tachycardia, which leads to episodic syncope, seizures and sudden death [52,53]. So far, two types of CPVT have been described based on their difference in the mode of inheritance. The autosomal dominant form that accounts for up to 50% of the cases has been linked to the mutations in the *RYR2* gene that encodes the cardiac ryanodine receptor [54], while a rare autosomal recessive form results from the mutations in the *CASQ2* gene that encodes the cardiac calsequestrin [55]. Functionally, ryanodine receptor and calsequestrin work together to mediate the release of calcium ions from the sarcoplasmic reticulum (SR) in the cardiac muscles during excitation-contraction coupling. As such, it is not surprising that mutations in the *RYR2* and *CASQ2* genes that result in functional derangements in intracellular calcium handling may result in arrhythmia. Since 2004, the pathophysiological roles of various *RYR2* and *CASQ2* mutations in driving the development of CPVT have been investigated in several transgenic animal or cell models [56,57,58,59]. Although most of the models were able to recapitulate the major CPVT phenotypes, such as SR calcium leak and catecholamine-induced delayed after-polarizations (DADs), the clinical significance of these models were limited by the substantial difference in the cardiac electrophysiology between rodents and human. Addressing this issue, the pathogenic effects of various CPVT associated mutations have been studied in patient-specific iPSCs models. These include the *RYR2*_F24831I_, *RYR2*_S406L_, *CASQ2*_D307H_ mutations [45,46,47]. Similar to the rodent models, all these iPSCs-based models were able to recapitulate the CPVT phenotypes, and the results confirmed that the diastolic SR calcium leak contributes to generation of DADs [45,46,47]. Except for disease modeling, the iPSCs-based CPVT models also provided a human cardiomyocytes-based platform for drug testing and toxicology studies. For example, in a recent report, Laugwitz and colleagues demonstrated that dantrolene ameliorates the CPVT phenotypes caused by *RYR2*_S406L_ mutation using the cardiomyocytes differentiated from the CPVT patient-specific iPSCs [46]. So far, only limited therapeutics, such as beta-blockers, are being used for treating CPVT. It is anticipated that the success in generating the CPVT-specific iPSCs may help facilitate the development of novel therapeutic approaches in treating CPVT.

### 5.2. Modeling Dilated Cardiomyopathy Associated with TNNT2 Mutation

Dilated cardiomyopathy (DCM) is the most common subtype of cardiomyopathy, and is characterized by the abnormal enlargement of ventricles, thinning of ventricular walls and the marked systolic dysfunction [33]. It has been estimated that about 50% of the cases are of genetic causes [60,61,62,63]. The pathological mechanisms associated with *TNNT2* gene mutations have been evaluated in a transgenic mouse model, in which the null mutation of this gene denoted an impaired contractile function of the heart [64,65]. Yet, how the specific *TNNT2* mutation contributes to the development of DCM phenotype in human remains ambiguous.

In 2012, Wu and colleagues generated iPSCs from DCM patients carrying a disease associated- mutation in the gene encoding cardiac troponin-T (*TNNT2*) [48]. Sequencing analysis showed that such mutation causes the 173rd amino acid residue of the cardiac troponin-T to change from an arginine (R) to a tryptophan (W). Clinically, patients with this mutation develop the typical DCM symptoms including left ventricle dilation and reduced ejection fraction. Skin biopsy samples were collected from affected and normal individuals of three generations of a single family for iPSCs generation. The resultant iPSCs were then differentiated into cardiomyocytes for functional analyses. When comparing to the control, the cardiomyocytes derived from the mutation containing-iPSCs showed abnormal sarcomeric alpha-actinin distribution. Functionally, the mutant cardiomyocytes exhibited impairments in contractility and reduction in calcium handling ability upon β-adrenergic stimulation. These observations indicated that the increased susceptibility to inotropic stress may be a signature characteristic of the *TNNT2*_R173W_ mutation in DCM development.

### 5.3. Modeling Cardiomyopathy Associated with DES Mutation

The *DES* gene encodes the intermediate filament protein desmin, but the exact function of desmin is not well defined. Nevertheless, mutations in the *DES* gene are commonly observed in DCM patients [66]. Phenotypically, mutations leading to the loss of *DES* gene function usually give rise to a significant accumulation of desmin-positive aggregates in the cardiomyocytes of affected individuals.

Lately, by utilizing the whole-exome sequencing approach, our laboratory has identified a novel *DES* mutation in a patient with left ventricular dilation and impaired left ventricular ejection function [49]. In this *DES* mutation, we recognized a change of the alanine (A) residue to valine (V) at the 285th amino acid position. In the transgenic mouse model with complete desmin deficiency, phenotypes such as hypertrophic and dilated cardiomyopathy [67] were observed. Surprisingly, the patient with *DES*_A285V_ mutation produced a mutant desmin possessing molecular weight and immunoreactivity comparable to the wild type desmin protein. To investigate the pathological significance of this novel *DES* mutation, we have generated skin fibroblasts-derived iPSCs from this *DES*_A285V_ patient. These *DES*_A285V_ iPSCs were subsequently differentiated into cardiomyocytes for structural and functional studies. When compared to the normal cardiomyocytes, the ones carrying the *DES* mutation exhibited abnormal protein aggregations in sarcomere and Z-disc streaming. In addition, contraction arrest was observed in the mutant cardiomyocytes upon isoproterenol stimulation. These observations not only provided an explanation to the pathogenic mechanism underlying the *DES*_A285V_ mutation, but also validated the causal ion relationship between the *DES* mutation and the DCM phenotype observed in that patient [49].

### 5.4. Modeling Hypertrophic Cardiomyopathy Associated with MYH7 Mutation

Hypertrophic cardiomyopathy (HCM) is a heritable cardiac disorder characterized by the abnormal left ventricular thickening and diastolic dysfunction in the absence of an identifiable hemodynamic cause [68]. About 13 HCM-associated genes have been identified to date and most of them encode sarcomeric proteins [69]. Transgenic mouse and rabbit models have been established for studying the pathological mechanisms of HCM [70,71,72]; however, the mechanistic roles of altered contractile function in the development of HCM remain inconclusive. Very recently, Lan and colleagues generated an iPSCs-line from patients carrying one HCM-associated mutation in the *MYH7* gene. In their case, the 663rd residue of the β-myosin heavy chain is changed from an arginine to a histidine as a result of a missense mutation. The patient-specific iPSCs were differentiated into cardiomyocytes for functional analyses. The mutant-containing cardiomyocytes recapitulated the key features of HCM, including increased cell size and arrhythmia. The intracellular calcium transient profile indicated that the diseased cardiomyocytes showed a significant increase in the resting intracellular calcium level when comparing to the normal cardiac muscle fibers. Interestingly, pharmaceutical inhibition of calcium entry helped to prevent the development of HCM phenotypes in the mutant cardiomyocytes suggesting the *MYH7* mutation altered the calcium homeostasis dysfunction [50].

### 5.5. Modeling Friedreich Ataxia Associated Cardiomyopathy

Apart from sarcomeric proteins, abnormality in the mitochondrial proteins may also contribute to HCM development. For example, deficiency in the mitochondrial protein frataxin may lead to Friederich ataxia (FRDA), in which patients usually develop with HCM phenotype to varying degrees [73]. In FRDA, abnormal expansions of the GAA repeat within the first intron of the *FXN* gene may result in the silencing of the gene, which in turn reduces or completely abolishes the production of the frataxin protein.

Frataxin has been implicated in the mechanism of iron-sulfur cluster biosynthesis; however, the contribution of frataxin-deficiency to cardiomyopathy development has yet to be elucidated.

To test whether iron homeostasis deregulation accelerates the reduction in energy synthesis dynamics that contributes to impaired cardiac calcium homeostasis and contractile force, we have recently generated skin fibroblasts-derived iPSCs from a FRDA patient [51]. The *FXN* gene expression in that patient was endogenously silenced. Phenotypically, the FRDA iPSCs*-*derived cardiomyocytes exhibited a disorganization of the mitochondrial network complemented with mitochondrial DNA depletion. Consistent with the mitochondrial disorganization, the energy synthesis dynamics, in terms of ATP production rate, in the diseased cardiomyocytes was impaired. Interestingly, when the diseased cardiomyocytes were subjected to iron overloading, a significant impairment in the calcium handling property was observed. These results indicated that patient-specific iPSCs are useful tools for studying FRDA-associated cardiac defects.

## 6. Application of Patient-Specific iPSCs-Derived Cardiomyocytes in Efficacy Testing and Drug Screening

Owing to the limited sources of human cardiomyocytes for *in vitro* analyses, the effects of a putative cardiac drug have to be conventionally tested in the well-established rabbit or canine Purkinje fiber model before proceeding to clinical trials. Nevertheless, as the non-human based cellular models often give false-positive or inconsistent results [74,75,76], many drugs that have passed the animal tests ended up with failure in the clinical trials. Recent reports demonstrated that human embryonic stem cells-derived cardiomyocytes exhibited excellent pharmacological response to various known antiarrhythmic agents; thus they may be a potential alternative to animal cardiomyocytes [77,78]. However, due to the difference in genetic background, individuals with similar cardiac disorders could show quite different responses towards a particular drug. In this regard, the patient-specific iPSC-derived cardiomyocytes offer an exclusive platform for evaluating the efficacy of a particular drug or treatment strategy on a personal basis.

Based on the latest breakthrough in the cardiac differentiation protocol, a yield of more than 80% in cardiomyocyte differentiation has been achieved [27]. When these patient-specific iPSCs-derived cardiomyocytes are applied to a high throughput assay platform, such as multielectrode arrays analysis, the effects of a testing drug on cellular electrophysiology can be evaluated in a short period of time. Emerging evidences from our group and other investigators have pointed out that altered calcium handling could be an important pathogenic mechanism underlying cardiomyopathies [48,49,79,80]; drugs that affect calcium homeostasis should be of great therapeutic potential. Mercola and colleagues have recently developed a high throughput automated kinetic image cytometry system for the measurement of calcium ion dynamics. This system enabled the authors to simultaneously measure individual calcium transients from 100 human iPSCs-derived cardiomyocytes [81]. Taking advantage of such system, high throughput screenings of calcium handling-enhancing properties of known or novel drugs can be performed on patient-specific iPSCs-derived cardiomyocytes.

## 7. Application of Patient-Specific iPSCs-Derived Cardiomyocytes in Toxicology Test

In addition to pharmacological studies, the cardiomyocytes derived from iPSCs are of great potential in the toxicology tests. So far, isolated canine cardiomyocytes are the most popular *pre*-clinical model for cardiac safety testing of a developing drug. However, as mentioned in the last section, the reliability of such model remains questionable. As a matter of fact, many drugs that have passed the animal tests turned out to show unanticipated cardiac toxicity when administered to patients [82], thus, a more predictive and reliable human cardiomyocyte-based model for toxicology test is of immediate demand. Increasing evidences suggested that the pharmacological sensitivities of human ESCs and iPSCs-derived cardiomyocytes are much more advanced than any animal models [77,78], and they should be good detectors for any undesired proarrhythmic side effects of a developing drug.

Recently, Mendenius and colleagues proposed the possibility of using human ESCs and iPSCs-derived cardiomyocytes in the evaluation of drug-induced cardiac injury [83,84]. In their studies, the human ESCs- and iPSCs-derived cardiomyocytes were treated with doxorubicin, and the release of cardiac troponin T in culture medium was measured utilizing a Biocore-based system for the degree of cell injury. Compared to the conventional ELISA based assay, the surface plasmon resonance-based method not only provides superior sensitivity and specificity, but also allows simultaneous analysis of multiple samples. Consequently, the use of iPSCs-derived cardiomyocytes in toxicity predication appears to be feasible.

## 8. Limitations of iPSCs

The recent achievement in the patient-specific iPSC technology has created a new platform for regenerative medicine, disease modeling and personalized medication development. Yet, like many other technologies, the clinical applications of patient-specific iPSCs-derived cardiomyocytes are also hindered by various limitations. Though the latest advancement in cardiac differentiation protocol allows efficient generation of cardiomyocytes in a high yield, the human iPSCs-derived cardiomyocytes are actually less mature in terms of calcium homeostasis when compared to the human ESCs-derived cardiomyocytes as demonstrated earlier by our laboratory [85]. In other words, the patient-specific iPSCs-derived cardiomyocytes may not be suitable for modeling cardiac defects resulted from mutations of genes that regulate calcium transients, such as the mutations in the gene encoding the phospholamban.

Furthermore, it should be noted that a high yield of cardiac differentiation is not equivalent to high purity. In fact, iPSCs-derived cardiomyocytes are always grown in a mixed population of atrial, ventricular and nodal subtypes. These subtypes do possess different electrophysiology properties. So far, most transplantation studies were performed in rodent models [28,29]. As rodents have a much faster heart rate compared to humans, the injection of human cardiomyocytes into rodent hearts may not create significant arrhythmia problems. However, the injection of mismatched subtypes of cardiomyocytes into a patient’s heart may lead to a medical emergency. Unfortunately, no efficient way is available to sort the subtypes of iPSCs-derived cardiomyocytes into pure populations. The direct application of patient-specific iPSCs cardiomyocytes in regenerative medicine, therefore, remains a theoretic foundation. Besides the issue of mixed subtypes, the immature phenotype of iPSCs-derived cardiomyocytes also limits its application in drug screening experiments. To this end, it is important to verify and validate the results obtained in the initial screening steps.

## 9. Conclusions

The cardiomyocytes derived from patients-specific iPSCs are of great potential in many clinical applications. This authentic human cardiomyocyte-based system is expected to compensate for the limitations of the current experimental animal models. This review provides detailed descriptions in the strategies and workflow of using the patient-specific iPSCs-derived cardiomyocytes in regenerative medicine, disease modeling and pharmacological applications. The examples illustrated in this review clearly evidenced the practical values of this novel technology. However, various limitations, such as the immaturities of iPSCs-derived cardiomyocytes, still need to be addressed, and future studies resolving these issues would be beneficial to the use of patient-specific iPSCs in clinical applications.
